# Added Value of Computed Tomography Angiography Prior to Bronchial Artery Embolization for Hemoptysis: A Retrospective Two-Center Study

**DOI:** 10.5334/jbsr.3097

**Published:** 2024-01-04

**Authors:** Paolo Ravetta, Michael Vouche

**Affiliations:** University Hospital Brugmann, Brussels, Belgium; Department of Radiology, Saint-Pierre University Hospital, Brussels, Belgium

**Keywords:** Bronchial artery embolization, CT-angiography, embolotherapy, hemoptysis, non-bronchial arteries

## Abstract

**Objectives:**

The aim of this retrospective study was to evaluate the added value of pre-procedural computed tomography angiography (CTA) prior to bronchial artery embolization for patients presenting with hemoptysis.

**Materials and Methods:**

In this retrospective study, we evaluated patients admitted for hemoptysis from 2010 to 2021 and treated by catheter-directed embolization. After establishing quality criteria for pre-procedural computed tomography (CT), patients were divided into two groups depending on their pre-procedural imaging assessment: Quality CT-angiography (QCTA group) and suboptimal pre-procedural imaging (suboptimal CTA, unenhanced or no CT evaluation; control group). Groups were compared based on radiological success, procedure-related complications, and clinical success, including cessation of hemoptysis, recurrence rates, and overall mortality.

**Results:**

We included 31 patients in the QCTA group, and 35 in the control group. Clinical success was *n* = 24/31 (77.4%) in the QCTA group and *n* = 27/35 (77.1%) in the control group (*p* = 0.979). Technical success was *n* = 37/42 (88.1%) in the QCTA group and *n* = 39/42 (92.86%) in the control group (*p* = 0.820). Overall recurrence was 10.6%. Minor complications occurred in 27.3%, and one major complication was reported.

The concordance between the affected bleeding lung and the identification of pathological arteries during angiography was better in the QCTA group (*p* = 0.045).

The average number of culprit arteries (bronchial, non-bronchial systemic arteries [NBSA] or pulmonary) in the QCTA group was not significantly higher than that in the control group.

**Conclusions:**

Preprocedural QCTA better identifies the affected bleeding lung and bleeding vessels compared to direct angiography. No difference in clinical success, complications, recurrence rates, or mortality was observed.

## Introduction

Bronchial artery embolization (BAE) is the standard treatment modality for controlling hemoptysis [1]. Due to its high invasiveness, surgery is limited to elective cases [2–5].

Bronchoscopy and chest computed tomography (CT) are considered the most reliable work-up methods for orienting the etiology and localizing the bleeding [2,4,6].

However, as CT can better visualize distal airways, lung parenchyma, and extrapulmonary structures such as vasculature, it is considered more effective than bronchoscopy for localizing the bleeding and identifying culprit vessels [2,6,7,8].

Hence, CT is currently considered the gold standard diagnostic tool for evaluating hemoptysis [1,2–5]. Recent technical improvements in contrast-enhanced computed tomography angiography (CTA) have dramatically improved the temporal and spatial resolution of CT reconstructions, increasing the detection of bronchial arteries, anatomical variants, or ectopic arteries, particularly non-bronchial systemic arteries (NBSA), which can be obscured during conventional angiography [1,2,5,7,9].

Although the use of CT for patients with hemoptysis is well documented [5,8], there are only a few established recommendations regarding pre-procedural bronchoscopy and CT protocol prior to BAE [2,5,6,8]. Despite its wide use in the diagnosis and management of critical conditions of hemoptysis, few studies have investigated objectively the added value of CTA for pre-treatment evaluation [8,10–12].

The aim of this retrospective study was to evaluate the added value of pre-procedural CTA prior to BAE for patients treated for hemoptysis in terms of technical success supported by procedural and CT data and clinical success, including complications, recurrence rates, and mortality.

## Materials and Methods

### Study population

This retrospective study was based on medical data collected from two university tertiary hospitals in Belgium, namely Erasmus and Saint-Pierre hospitals. We submitted and obtained approved consent from each hospital’s ethics committee.

We included every patient admitted from January 2010 to January 2021 for therapeutic thoracic angiography in cases of hemoptysis.

Based on recent guidelines on CTA definition [2], we established the following quality criteria for pre-procedural CT: (1) contrast-enhanced CT; (2) acquisition timed to coincide with peak arterial enhancement depending on the suspected etiology of hemoptysis (pulmonary arteries vs. aorta vs. both (mixed phase or sequential acquisition); (3) thin-section acquisitions <2.0 mm; (4) visualization of the vascular anatomy.

Two authors (Paolo Ravetta and Michael Vouche) retrospectively reviewed every patient’s pre-procedural CT.

CT interpretation focused first on specific parenchymal abnormalities (e.g., bronchiectasis, ground glass opacity, cavitary lesions, infiltration) and other lesions such as bronchial tree filling defects, masses, or pathological vessels to confirm the underlying etiology and discern the affected bleeding lung in right, left, bilateral lung bleeding, or non-visible bleeding. Secondly, we focused on the number, location, diameter, and course of bronchial arteries and NBSA, searching for pathological vessels.

Bronchial arteries included orthotopic bronchial arteries originating from the levels of T5 and T6 vertebrae and ectopic bronchial arteries from any level of the aorta except levels T5 and T6 vertebrae, or its branches.

Bronchial arteries were considered abnormal when their diameter was ≥2 mm or their courses were tortuous. NBSAs were considered abnormal if they were dilated and tortuous within extra pleural fat related to pleural thickening.

Disagreements in quality assessment were settled by consensus. Applying quality criteria, patients were divided into two groups depending on their pre-procedural CT: quality CT-Angiography (QCTA group) and suboptimal CTA, unenhanced or no CT evaluation (control group).

Massive hemoptysis was classified in line with the previous definition [13].

Patients’ data were obtained from medical records from the time of admission to the date of discharge. Primary outcomes included technical success supported by procedural and CT data. Secondary outcomes were clinical success, complications, recurrence rates, and mortality.

### Imaging

Given the duration of the period of investigation, different CT scanners and protocols were used. Our current CTA protocol is performed with a 128- or 384-detector row scanner (Somatom Definition or Force, Siemens, Erlangen, Germany) with a scan range from the apex of the lung to its base.

Approximately 80 to 100 mL of contrast agent (Omnipaque 300 mgI/mL; GE Healthcare, Oslo, Norway) is injected at 4 mL/s through the antecubital vein, followed by 30 mL (2 mL/s) of saline solution.

Automatic bolus-triggering software is used, with a circular region of interest positioned at the level of the pulmonary trunk for pulmonary artery evaluation and at the level of the descending aorta for systemic artery evaluation. Post-processing is performed on conventional dedicated software.

### BAE procedure

Angiography was performed under local or general anesthesia depending on the condition of the patient, favoring vascular femoral access using a 4 or 5 French introducer sheath. Aortography was performed when needed using a pigtail catheter. Pathological vessels were catheterized using 4 or 5 French catheters, and 2.0 to 2.7 French microcatheters were used when necessary for superselective catheterization.

Preferred embolic agents were non-resorbable microparticles 300 to 500 or 500 to 700 µm; Polyvinyl alcohol (PVA) (DC-Bead; Terumo Medical Corporation, Tokyo, Japan), and Microspheres (Boston Scientific, Marlborough, Massachusetts, USA). Used alone or in combination with other embolic materials such as Gelfoam (Pfizer, Manhattan, New York, USA), n-Butyl cyanoacrylate (NBCA) (Histoacryl; B. Braun Melsungen AG, Melsungen, Germany) and Coils (Tornado, Cook Medical, Bloomington, Indiana, USA; AZUR, Terumo Medical Corporation, Tokyo, Japan). Amplatzer (Micro Vascular Plug; Medtronic, Dublin, Ireland) was used for a ruptured pulmonary artery.

The choice of embolic agents was made based on operators’ discretion, according to the location of the pathological vessel, and on operators’ habits.

Technical success was defined as the ability to cannulate and embolize all visualized abnormal arteries in the involved lung segments. Clinical success was defined as a complete cessation or clinically reduced hemoptysis within 24 hours of the BAE or during the same admission period. Recurrence rates were defined as clinically significant hemoptysis occurring after the BAE, requiring medical management, repeated embolization, or surgery.

BAE complications were divided into minor or major and classified according to the Society of Interventional Radiology guidelines [14].

### Statistical analysis

Baseline characteristics between groups were compared using the two-sided Chi-square test or Mann–Whitney U test for categorical variables and continuous variables, respectively. A *p <* 0.05 was defined as statistical significance.

## Results

### Patient characteristics

Sixty-six patients fulfilled inclusion and exclusion criteria*.* Thirty-one patients composed the QCTA group, and 35 patients composed the control group. Demographic and etiology are presented in Table 1.

**Table 1:** Patient characteristics.

	**QCTA (*N* = 31)**	**CONTROL GROUP (*N* = 35)**	**TOTAL (*N* = 66)**	***P* VALUE**
Age* **(years)**	50.00 ± 18.23	57.71 ± 17.85	54.09 ± 18.31	0.071
**Male/female ratio**	26/5	26/9	52/14	0.346
**Hemoptysis amount**				
Massive	14 (45.2%)	9 (25.7%)	23 (34.8%)	0.165
Non-massive	17 (54.8%)	26 (74.3%)	43 (65.2%)	
**Pre-procedural bronchoscopy**	18 (58.1%)	23 (65.7%)	41 (62.1%)	0.526
**Etiology**				
Bronchiectasis	4 (12.9%)	3 (8.6%)	7 (10.6%)	0.571
Tuberculosis	4 (12.9%)	2 (5.7%)	6 (9.1%)	0.314
Aspergilloma	1 (3.2%)	4 (11.4%)	5 (7.6%)	0.212
Malignancies	6 (19.3%)	9 (25.7%)	15 (22.7%)	0.542
Infections	4 (12.9%)	3 (8.6%)	7 (10.6%)	0.571
Cystic fibrosis	4 (12.9%)	1 (2.9%)	5 (7.6%)	0.127
Other^a^	6 (19.3%)	6 (17.1%)	12 (18.2%)	0.818
SARS-COV-2	1 (3.2%)	4 (11.4%)	5 (7.6%)	0.212
Idiopathic/cryptogenic	1 (3.2%)	3 (8.6%)	4 (6.1%)	0.367
**Transfer**	16 (51.6%)	18 (51.4%)	34 (51.5%)	0.988

*SARS-COV-2*, *s*evere acute respiratory syndrome coronavirus 2.

* Data are mean ± standard deviation.

a “Other” includes: Pulmonary hypertension (*n* = 2), Behcet (*n* = 1), atelectasis (*n* = 1), pulmonary pseudo-aneurysm (*n* = 1), Wegener disease (*n* = 1), vascular arterio-artery malformation (*n* = 1), iatrogenic post-biopsy (*n* = 1), acute pulmonary embolism (*n* = 1), chronic thromboembolic disease (*n* = 1), Anthraco-silicosis (*n* = 1), chronic obstructive pulmonary disease (*n* = 1).

### Pre-procedural CT examination

Most patients in the control group had contrast-enhanced CT *n* = 23 (65.7%) (considered suboptimal), ten patients had unenhanced CT (28.6%), and two had no pre-procedural CT (5.7%) (Table 2).

**Table 2:** Pre-procedural CT.

	**QCTA (*N* = 31)**	**CONTROL GROUP (*N* = 35)**	**TOTAL (*N* = 66)**	***P* VALUE**
CT with IV C+	31 (100%)	23 (65.71%)	54 (81.81%)	
CT	NA	10 (28.57%)	10 (15.15%)	
none	NA	2 (5.71%)	2 (3.03%)	
Bleeding site				0.214
Right	17 (54.84%)	12 (34.28%)	29 (43.94%)	
Left	4 (12.90%)	6 (17.14%)	10 (15.15%)	
Bilateral	10 (32.26%)	11 (31.43%)	21 (31.82%)	
Not visible	0 (0.0%)	3 (8.57%)	3 (4.54%)	
No data available	0 (0.0%)	3 (8.57%)	3 (4.54%)	

NA, Not applicable.

Based on CT evaluation, we divided the affected bleeding lung into right, left, bilateral lung bleeding, or non-visible bleeding, and a comparison between CT and angiography was performed.

The concordance between the affected bleeding lung and the identification of pathological arteries during angiography was concordant in 24/32 (75.0%) patients in control group and in 29/31 (93.54%) patients in the QCTA group (*p* = 0.045).

### Angiographic evaluation and embolization

During angiographic procedures, the decision to proceed with embolization was made in 26 (83.9%) patients in the QCTA group and in 31 (88.6%) patients in the control group.

Non-embolization was decided for nine patients in the absence of detection of bronchial arteries (*n* = 4) and a normal appearance of bronchial arteries with clinical improvement (*n* = 5).

The average number of culprits NBSAs was similar between the QCTA group and the control group (9 vs. 2, *p* = 0.102) (Table 3). The average number of culprit bronchial arteries (*p* = 0.254) and culprit pulmonary arteries (*p* = 0.294) did not differ between groups. No significant difference was observed in vascular access (venous, arterial, or both) (*p* = 0.136) or laterality of embolization (*p* = 0.782). The total number of pathologic arteries (*p* = 0.520) and technical success was similar in both groups (88.1% vs. 92.9%, *p* = 0.820). Angiographic evaluation data are shown in Table 4.

**Table 3:** Culprit arteries identified through CT and angiographic evaluation**.**

**NUMBER OF CULPRIT ARTERIES**	**QUALITY CTA (*N* = 31)**	**CONTROL GROUP (*N* = 35)**	**TOTAL (*N* = 66)**
**Bronchial arteries**			
Right BA	16	19	35
Left BA	11	13	24
Common trunk	1	2	3
RICBT	1	3	4
LICBT	0	2	2
Total	29	39	68 (80.95%)
**Culprit NBSAs**			
Aortic arch	1	0	1
Subclavian	3	1	4
Internal mammary	4	0	4
Intercostal	1	1	2
Total	9	2	11 (13.09%)
**Pulmonary arteries**	4	1	5 (5.95%)
**Total culprit arteries**	42	42	84

*RICBT,* Right intercostal bronchial trunk; *LICBT*, Left intercostal bronchial trunk.

**Table 4:** Angiographic data.

	**QCTA (*N* = 31)**	**CONTROL GROUP (*N* = 35)**	**TOTAL (*N* = 66)**	***P* VALUE**
**Decision to proceed with embolization**	26 (83.8%)	31 (88.6%)	57 (86.4%)	0.582
Arterial	24 (77.4%)	33 (94.3%)	57 (86.4%)	
Venous	3 (9.7%)	1 (2.9%)	4 (6.1%)	
Arterial + venous	4 (12.9%)	1 (2.9%)	5 (7.6%)	
**Embolization site**				0.782
Right	16 (51.6%)	16 (45.7%)	32 (48.5%)	
Left	6 (19.3%)	8 (22.9%)	14 (21.2%)	
Bilateral	3 (9.7%)	5 (14.3%)	8 (12.1%)	
**Embolic material**				
PVA alone	18	22	40	
PVA + Gelfoam	1	0	1	
PVA + *N*-butyl-cyanoacrylate	0	1	1	
PVA + coils	1	0	1	
PVA + coils +Gelfoam	2	1	3	
Coils alone	3	0	3	
Gelfoam alone	0	3	3	
Gelfoam + coils	0	1	1	
Amplatzer plug	0	1	1	
**Technical success**	88.10%	92.86%	90.48%	0.820
**Total no. arteries embolized**	37/42	39/42	76/84	

### Clinical success and recurrence rates

Minor post-procedure complications were seen in 18 (27.3%) patients (*n* = 11; 35.5% vs. *n* = 8; 28.8%, *p* = 0.269). One patient suffered from lower limb ischemia after three embolization sessions and femoral artery dissection.

Clinical success was similar in both groups (*n* = 24; 77.4% vs. *n* = 27; 77.1%, *p* = 0.979). Of the seven patients without clinical success in the QCTA group, three had persistent hemoptysis and underwent a second BAE to achieve clinical success; two patients suffered recurrence and required a second BAE; and two patients suffered recurrence of massive lethal hemoptysis. Of the eight patients without clinical success in the control group; four had persistent hemoptysis and underwent a second BAE (*n* = 2), surgery, and palliative care, respectively; two suffered mild hemoptysis recurrence and underwent a second BAE; and one suffered massive lethal hemoptysis recurrence.

Mortality during hospitalization was seen in 20 patients (30.3%) (*n* = 9; 29.0% vs. *n* = 11; 30.3%, *p* = 0.425), mainly from disease progression and comorbidities; only three died from the recurrence of massive hemoptysis (4.5%).

Outcomes and complications are shown in Table 5.

**Table 5:** Outcomes.

**PARAMETER**	**QUALITY CTA (*N* = 31)**	**CONTROL GROUP (*N* = 35)**	**TOTAL (*N* = 66)**	***P* VALUE**
**Post-procedural complications**				0.269
Minor	11 (35.5%)	8 (22.9%)	19 (28.8%)	
Major	—	1 (2.9%)	1 (1.5%)	
Total	11 (35.5%)	9 (25.7%)	20 (30.3%)	
**Clinical success**	24 (77.4%)	27 (77.1%)	51 (77.3%)	0.979
**Recurrence**	4 (12.9%)	3 (8.6%)	7 (10.6%)	0.571
**Minor complications**				
Thoracic pain	3	4	7	
Fatigue	2	1	3	
Dysphagia	—	1	1	
Cough	5	1	5	
Dyspnea	2	2	4	
Sore throat	—	2	2	
Total	12	11	23	
**Major complications**				
Lower limb ischemia due to thrombus	—	1	1	
**Repeated BAE**	6 (19.3%)	6 (17.1%)	12 (18.2%)	0.818
**Cause of mortality during hospitalization**				0.425
Massive hemoptysis	2 (6.4%)	1 (2.9%)	3 (4.5%)	
Other causes	7 (22.6%)	10 (28.6%)	17 (25.8%)	
Total	9 (29.0%)	11 (31.4%)	20 (30.3%)	

## Discussion

Many studies on BAE for hemoptysis have been published in the last decade, mainly from Asia, where tuberculosis remains a dominant etiology.

In comparison to the main relevant studies on BAE, including the systematic review by Panda et al. [13], the prevalence of diseases with a high risk of recurrence, such as lung malignancies (*n* = 15; 22.7%), aspergilloma, and cystic fibrosis [1,13,15] was higher in our study. Particularly, a lower clinical success rate in lung malignancies compared to other etiologies has been reported in dedicated studies [16,17]. This can explain the lower clinical success rate in our study (77.3%) compared to most recent studies, despite an overall technical success (90.5%) and recurrence rate during hospitalization (10.6%) in line with previous studies [1,12,13,18,19].

CT is commonly considered the gold standard diagnostic tool for evaluating hemoptysis [10]. Interestingly, our study demonstrated that pre-procedural CT evaluation for the site of bleeding lung was more accurate in QCTA group compared to direct angiography (*p* = 0.045), supporting the added value of optimal protocols of contrast injection, acquisition conditions, and reconstruction techniques.

**Figure 1:**
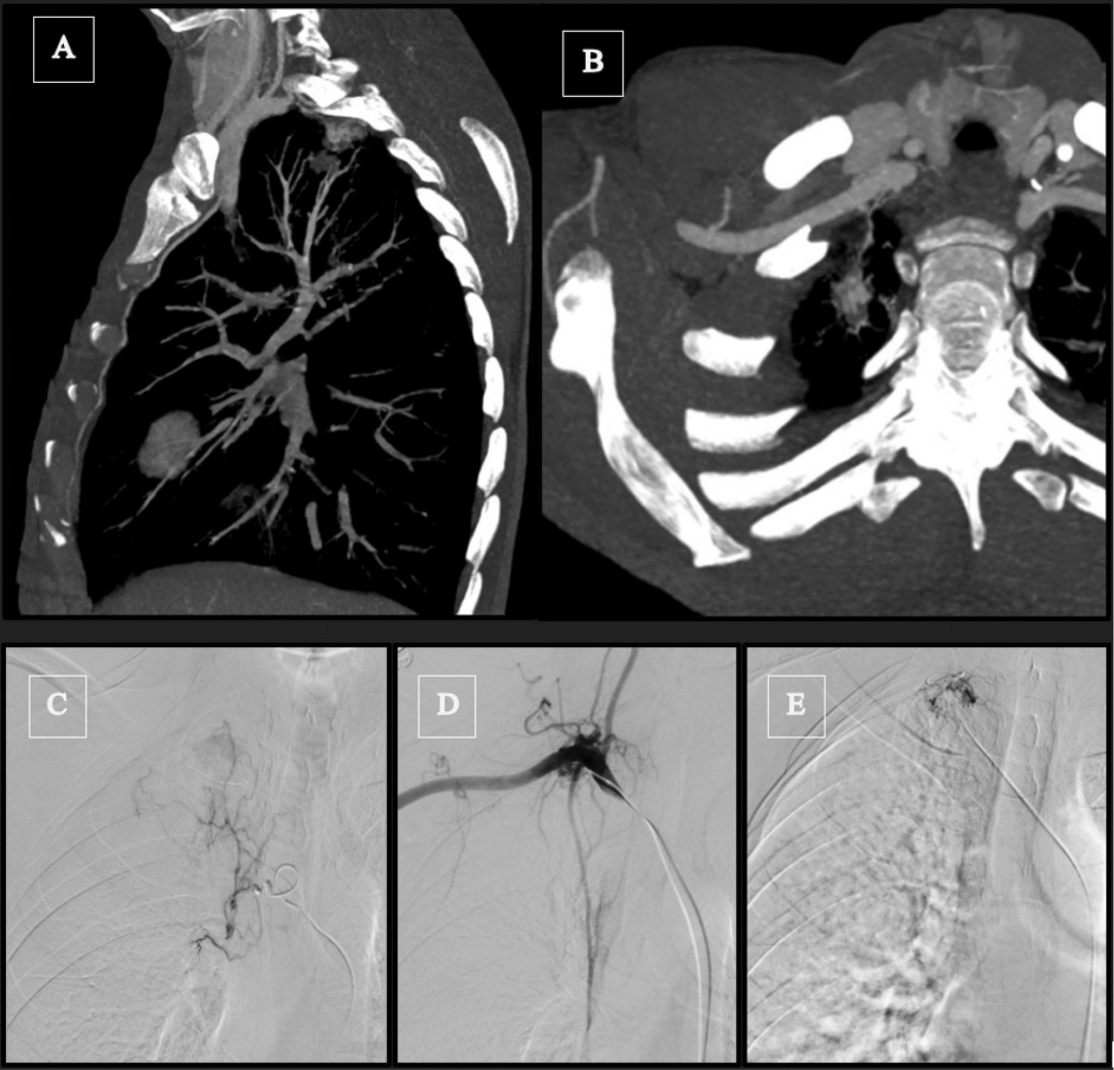
Forty-year-old patient with a right upper lobe arteriovenous malformation (AVM) or vascular degeneration mimicking AVM post-tuberculosis sequelae. (A, B) Angiography CT sagittal maximum intensity projections (MIP) and volume rendering technique (VRT) showing a vascular nidus located in the right upper lobe with the participation of a NBSA coming from the subclavian artery. (C) Angiography of the right bronchial artery shows blushing at the lesion site. (D, E) Angiographic catheterization and embolization of the NBSA coming from the subclavian artery.

By better depicting the pathological vasculature, CTA helps the interventional radiologist optimize the embolization plan by refining the origin of culprit vessels, precising orientation and tortuosity of vessels, and improving the choice of vascular access and the choice of (micro)catheter(s) and embolic materials ([11,12, Figure 1]).

Two prospective studies demonstrated that even in emergency settings, CTA provides useful information likely to change patient management, especially in the treatment of choice and in the vascular approach (pulmonary vs. aortic vs. both) [10,11] and could improve hemoptysis-free early survival while preventing future interventions [12].

In our study, no difference in clinical success, technical success, post-procedure complication and recurrence rates during hospitalization was observed between the two groups. This might be explained by the retrospective design of the study, the small sample size, and by the complex physiopathology of hemoptysis. Also, a risk of selection bias is possible as part of the control group, even if deemed “suboptimal” received contrast-enhanced CT (65.7%) that could also have influenced the pre-procedural planning.

Recurrence during hospitalization was 10.6%, and repeated angiographic evaluation with embolization was performed in 19.7% of patients. The main etiologies for recurrence reported in the literature are vessel recanalization, neo-angiogenesis, previous technical failure, or incomplete embolization [3,5,20].

In our study, partial embolization was the most prevalent cause, especially in the control group. This could indicate that, as mentioned before, pre-procedural CTA helped the interventional radiologist visualize and embolize all pathological vessels, especially NBSA or ectopic arteries.

Considering the identification of culprit NBSA, pulmonary arteries involvement and the choice of vascular access (alone or combined with arterial) in our study, the trend was in favor of the QCTA group. Indeed, trends of higher number of culprits NBSA, pulmonary artery, and femoral vein approaches in the QCTA group than in the control group, which could also indicate the orientation of treatment angiography by QCTA.

This study is inherently subject to the limitations of a retrospective study. The data reported here are the data available on medical charts. The relatively small sample size and limited data may have affected statistical power and, therefore, statistical significance.

In conclusion, QCTA was found to better identify the affected bleeding lung and bleeding vessels compared to direct angiography, suggesting that in cases of hemoptysis, pre-procedural CT-angiography may depict better ectopic arteries, NBSA, and pulmonary artery involvement.

However, we failed to demonstrate an impact of QCTA on clinical success, complications, recurrence rates, or mortality. Additional studies, ideally prospective, should be conducted to further examine and validate the methodology and the clinical impact of CTA as an image work-up prior to BAE.
